# *Escherichia coli* O157:H7 Outbreak Associated with Seasonal Consumption of Raw Ground Beef — Wisconsin, December 2012–January 2013

**Published:** 2013-12-06

**Authors:** Carol Quest, Rachel Klos, Jeffrey P. Davis, Abbey J. Canon

**Affiliations:** Watertown Dept of Health; Wisconsin Div of Public Health; EIS Officer, CDC

On January 8, 2013, the Wisconsin State Laboratory of Hygiene notified the Wisconsin Division of Public Health (WDPH) of two patients with *Escherichia coli* O157:H7 clinical isolates that had indistinguishable, but commonly identified, pulsed-field gel electrophoresis (PFGE) patterns. The two patients were interviewed by local health departments within 1 day of the initial report. They revealed that they had eaten raw ground beef purchased from the same meat market and served as “tiger meat” or “cannibal sandwiches.” In this dish, the raw ground beef typically is served on rye bread or crackers with onions and is a traditional winter holiday specialty in certain regions of the upper Midwest. Five agencies (the Watertown Department of Health; WDPH; Wisconsin Department of Agriculture, Trade, and Consumer Protection; U.S. Department of Agriculture’s Food Safety and Inspection Service; and CDC) investigated to determine the magnitude of the outbreak, prevent additional infections, and better understand raw ground beef consumption.

The market provided a list of 62 persons who preordered raw ground beef for the 2012 winter holiday season. A case-finding and knowledge-attitudes-practices questionnaire was administered to 53 of 62 persons included on that list, plus nine additional household members, and two persons with reported illness. A probable case was defined as diarrhea with onset occurring in a person who had been exposed in the previous 10 days to raw ground beef sold by the market during December 22, 2012–January 4, 2013. A confirmed case was an illness meeting the probable case definition in a person from whose stool *E. coli* O157:H7 with PFGE and multilocus variable-number tandem-repeat analysis (MLVA) patterns indistinguishable from those of the outbreak strain had been isolated.

Among 17 patients (four with confirmed and 13 with probable cases), 13 were female, and median age was 46 years (range: 1–82 years). Eight (47%) had received outpatient medical care; no hospitalizations or deaths occurred. Fourteen patients reported eating raw ground beef served as tiger meat or cannibal sandwiches during the holiday, and three had exposure to raw ground beef from cross-contamination. The market voluntarily recalled 2,532 pounds (1,148 kg) of raw ground beef on January 15, 2013. *E. coli* O157:H7 isolates from four patients and two raw ground beef samples (one in original packaging) collected from two households had PFGE and MLVA patterns indistinguishable from the outbreak strain.

Among respondents to the questionnaire, 55 (98%) of 56 reported consuming raw ground beef only during special occasions or winter holidays. A total of 53 (91%) of 58 were aware that consuming raw ground beef could cause illness, but only 17 (41%) of 42 thought that illness could be severe. Six of 15 (40%) patients and 28 (70%) of 40 non-ill persons said they intended to eat raw ground beef in the future.

In this same region of Wisconsin, raw ground beef served as tiger meat was associated with large (more than 50 cases) outbreaks of foodborne illness reported to WDPH during 1972, 1978, and 1994 (1–3). Despite ongoing outreach efforts addressing the dangers associated with consuming undercooked or raw ground beef, this regional holiday tradition continues to be associated with outbreaks.

Epidemiologic, laboratory, and traceback evidence implicated raw ground beef from the market as the source of *E. coli* O157:H7 in this outbreak. The rapid public health response resulted in timely case detection and likely prevention of additional cases through product recall.

Discouraging this tradition requires regional targeted consumer and retailer education to ensure understanding of the potential for severe illness associated with raw ground beef consumption. Retailers in this region should be encouraged to directly discourage their customers from consuming raw ground beef. To prevent illness, ground beef should be cooked to an internal temperature of 160°F (71°C), as measured with a food thermometer, before consumption.
